# Competitively disrupting the neutrophil-specific receptor–autoantigen CD177:proteinase 3 membrane complex reduces anti-PR3 antibody-induced neutrophil activation

**DOI:** 10.1016/j.jbc.2022.101598

**Published:** 2022-01-19

**Authors:** Stephen F. Marino, Uwe Jerke, Susanne Rolle, Oliver Daumke, Ralph Kettritz

**Affiliations:** 1 Experimental and Clinical Research Center, Max Delbrück Center for Molecular Medicine in the Helmholtz Association (MDC), Charité - Universitätsmedizin Berlin, Germany; 2 Structural Biology of Membrane-Associated Processes, MDC, Berlin, Germany; 3 Nephrology and Intensive Care Medicine, Charité Campus Virchow, Berlin, Germany

**Keywords:** neutrophil, PR3, ANCA, CD177, activation, 7-AAD, 7-actinomycin D, ANCA, antineutrophil cytoplasmic autoantibody, Ann V, annexin V, FACS, fluorescence-activated cell sorting, IgG, immunogloblin G, MACS, magnetic-activated cell sorting, mPR3, membrane-bound PR3, mPR3^low^, low amounts of mPR3, mPR3^high^, high amounts of mPR3, MWCO, molecular weight cutoff, PR3, proteinase 3, RU, resonance unit, SPR, surface plasmon resonance, TNFα, tumor necrosis factor alpha

## Abstract

CD177 is a neutrophil-specific receptor presenting the proteinase 3 (PR3) autoantigen on the neutrophil surface. CD177 expression is restricted to a neutrophil subset, resulting in CD177^pos^/mPR3^high^ and CD177^neg^/mPR3^low^ populations. The CD177^pos^/mPR3^high^ subset has implications for antineutrophil cytoplasmic autoantibody (ANCA)–associated autoimmune vasculitis, wherein patients harbor PR3-specific ANCAs that activate neutrophils for degranulation. Here, we generated high-affinity anti-CD177 monoclonal antibodies, some of which interfered with PR3 binding to CD177 (PR3 “blockers”) as determined by surface plasmon resonance spectroscopy and used them to test the effect of competing PR3 from the surface of CD177^pos^ neutrophils. Because intact anti-CD177 antibodies also caused neutrophil activation, we prepared nonactivating Fab fragments of a PR3 blocker and nonblocker that bound specifically to CD177^pos^ neutrophils. We observed that Fab blocker clone 40, but not nonblocker clone 80, dose-dependently reduced anti-PR3 antibody binding to CD177^pos^ neutrophils. Importantly, preincubation with clone 40 significantly reduced respiratory burst in primed neutrophils challenged with either monoclonal antibodies to PR3 or PR3–ANCA immunoglobulin G from ANCA-associated autoimmune vasculitis patients. After separating the two CD177/mPR3 neutrophil subsets from individual donors by magnetic sorting, we found that PR3–ANCAs provoked significantly more superoxide production in CD177^pos^/mPR3^high^ than in CD177^neg^/mPR3^low^ neutrophils, and that anti-CD177 Fab clone 40 reduced the superoxide production of CD177^pos^ cells to the level of the CD177^neg^ cells. Our data demonstrate the importance of the CD177:PR3 membrane complex in maintaining a high ANCA epitope density and thereby underscore the contribution of CD177 to the severity of PR3–ANCA diseases.

As the most abundant leukocytes, neutrophil granulocytes represent one of the first lines of defense against infectious agents and are therefore a pillar of the innate immune system. Among their most potent defense mechanisms are the respiratory burst to generate reactive oxygen species and degranulation, whereby stores of cytotoxic species housed in several types of intracellular and membrane-bound compartments called granules, are moved to the cell surface and released into the extracellular environment as a response to pathogen detection (1). This toxic cocktail is designed to kill foreign cells in the vicinity of the neutrophil. Given that healthy cells are also negatively affected, degranulation is a highly regulated process (though not yet fully understood) (2). The serine protease proteinase 3 (PR3) is found in large abundance in human neutrophils (3). It is a major component of neutrophil azurophilic granules but is interestingly also detectable on the outer surface of the neutrophil plasma membrane. In most individuals, two distinct neutrophil populations can be identified based on the amount of membrane-bound PR3 (mPR3) they harbor—one with low amounts of mPR3 (mPR3^low^) and another with orders of magnitude more detectable mPR3 (mPR3^high^) (4). The mPR3^high^ population is further distinguished by the presence of a selectively expressed membrane receptor called CD177 (5, 6). CD177 is a glycosylphosphatidylinositol-anchored protein exclusively expressed in a subset of neutrophils and forms a high-affinity complex with PR3. It thus accounts for the increased mPR3 levels that are detectable on the mPR3^high^ subset (7). The proportion of CD177^pos^/mPR3^high^ *versus* CD177^neg^/mPR3^low^ neutrophils in a given individual is genetically determined and remains constant throughout life (8, 9, 10). Although the function of CD177 is still unclear, several studies have identified a correlation between a large CD177^pos^/mPR3^high^ neutrophil population and the occurrence and progression of a group of incurable autoimmune diseases called antineutrophil cytoplasmic antibody (ANCA) vasculitides (8, 11, 12, 13, 14). In these disorders, autoantibodies directed against PR3 stimulate respiratory burst and degranulation. The resulting release of reactive oxygen species and cytotoxic enzymes and peptides—circumventing the normally strictly controlled degranulation process—causes considerable systemic damage to healthy tissue and is the hallmark of these conditions. It has been shown that, although all neutrophils are activated upon exposure to PR3–ANCAs, CD177^pos^/mPR3^high^ neutrophils react more strongly to autoantibody binding, as measured by degranulation, generation of superoxide (an initial product of the respiratory burst—referred to as “oxidative burst”), and increased phosphorylation of Akt kinase (15). AAV patients with large CD177^pos^/mPR3^high^ populations are more prone to relapse and show poorer clinical outcomes than those with smaller CD177^pos^/mPR3^high^ populations (11, 12, 13).

The mechanism by which PR3–ANCAs cause neutrophil activation is not known. Since all neutrophils display mPR3 and are affected by PR3–ANCAs, the presence of PR3 seems critical for the process. In the case of CD177^pos^/mPR3^high^ neutrophils, which are more strongly affected by the binding of PR3–ANCAs, the questions arise whether and how CD177 itself may contribute to ANCA-stimulated degranulation. Although CD177 does not cross the plasma membrane, it could interact with other species that do and in this way enhance the sensitivity of CD177^pos^/mPR3^high^ neutrophils to the effects of PR3–ANCAs.

We sought to directly test the contribution of CD177 to PR3–ANCA-stimulated neutrophil activation. To this end, we generated a series of anti-CD177 antibodies, some of which bound to the CD177:PR3 complex and some of which blocked the binding of PR3. We used Fab fragments derived from the latter to selectively disrupt CD177:PR3 complexes on CD177^pos^/mPR3^high^ neutrophils. We then tested the effect of this treatment on PR3–ANCA-induced respiratory burst using both mixed and sorted neutrophil populations. We show that removing CD177-bound PR3 reduces the sensitivity of mixed neutrophil pools to PR3–ANCA treatment. When we tested separated CD177^neg^/mPR3^low^ and CD177^pos^/mPR3^high^ populations, we found that while Fab treatment had no effect on the PR3–ANCA-induced respiratory burst of CD177^neg^/mPR3^low^ neutrophils, the anti-CD177 Fabs reduced the response of the CD177^pos^/mPR3^high^ population to that of the CD177^neg^/mPR3^low^ population. Thus, the excess mPR3 on CD177^pos^/mPR3^high^ neutrophils appears to account for their enhanced sensitivity to PR3–ANCAs. The presence of CD177 enables a higher density of PR3 epitopes that result in a stronger activation effect in response to autoantibody binding than seen in CD177^neg^ neutrophils.

## Results

### Screening of anti-CD177 monoclonal antibodies identifies binders that block the CD177:PR3 interaction

**Figure 1:**
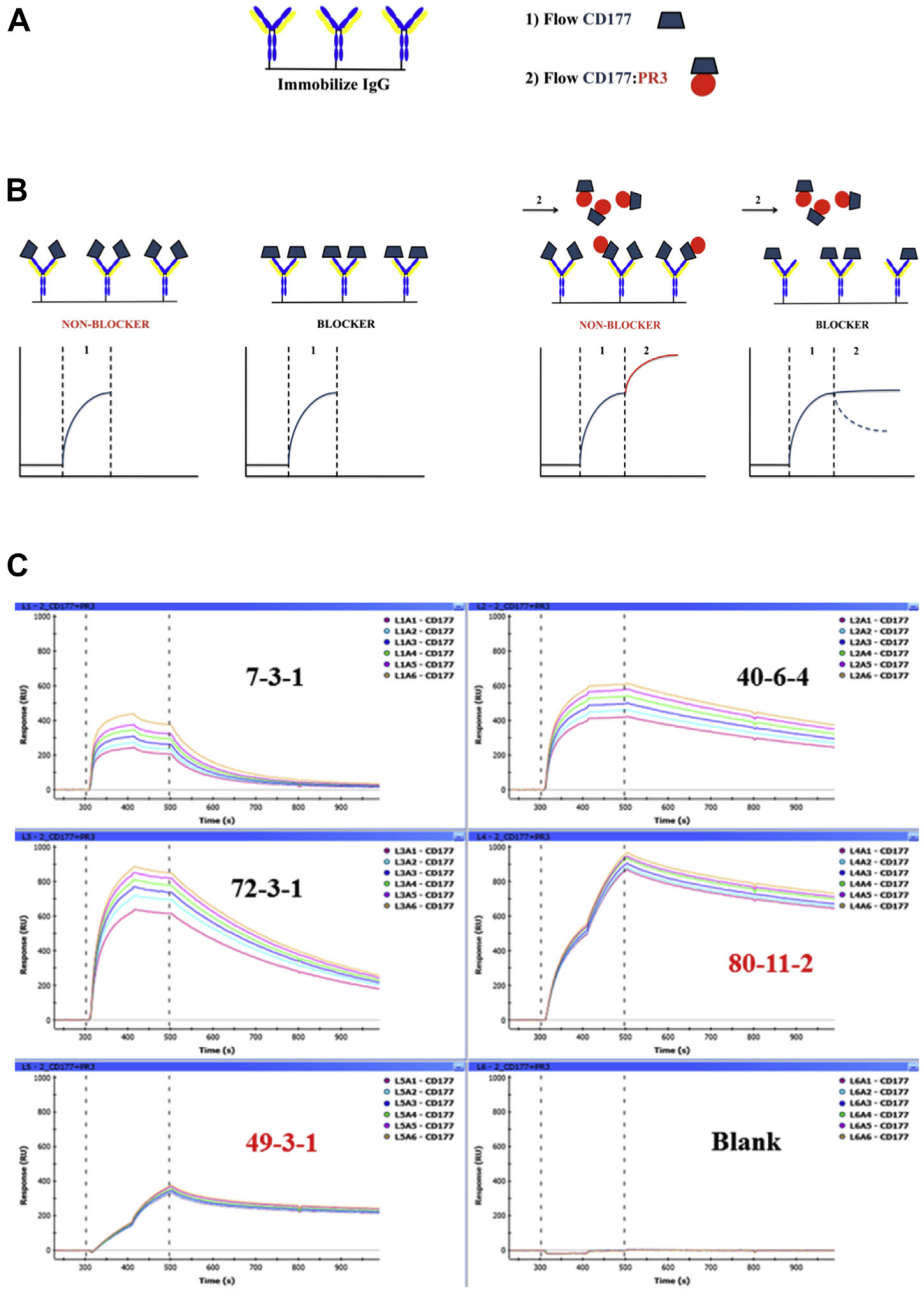
**Identification of anti-CD177 blocker IgGs by SPR.***A*, IgG was immobilized on the SPR sensor chip and subjected to two consecutive ligand flows—first with CD177 alone and second with the CD177:PR3 complex. *B*, expected sensorgrams for each ligand flow for both blocker and nonblocker IgG; *left panel*, ligand flow 1; *right panel*, ligand flow 2. *C*, SPR sensorgrams showing blocker (labeled in *black*) and nonblocker (labeled in *red*) IgG; ligand flows 1 and 2 occurred between the *horizontal dotted lines*. IgG, immunoglobulin G; SPR, surface plasmon resonance.

We used recombinant CD177 (7) for the generation of mouse monoclonal antibodies against CD177. Ten of the resulting hybridoma products were assessed by surface plasmon resonance (SPR) spectroscopy to determine their binding affinities for CD177. All 10 immunogloblin Gs (IgGs) bound CD177 with high affinity, ranging from 0.14 to 11.9 × 10^−9^ M (Table 1). We attempted to determine the ability of each IgG to block the binding of PR3 to CD177 by competition ELISA using an anti-PR3 antibody but were unable to optimize the assay to produce unambiguous results. We therefore devised a direct assay using SPR as depicted in Figure 1. We immobilized each anti-CD177 IgG to the sensor chip and subjected them to two back-to-back ligand flows: the first ligand sample contained only CD177, which was allowed to flow over the immobilized IgGs until the resonance units (RUs) indicated near saturation of all binding sites (Fig. 1, *A* and *B*); the second ligand sample containing CD177:PR3 complexes was then injected, and the resulting effect on the RUs was observed. Two possible RU responses could be expected. If the IgG in question does not interfere with the PR3 interaction with CD177 (*i.e.*, the IgG was a *nonblocker*), then CD177:PR3 complexes could be exchanged for CD177 in the IgG-binding sites, leading to a second pronounced increase in the RUs as the extra mass from PR3 was added to each binding interaction (Fig. 1*B*). Conversely, if the IgG does prevent interaction of PR3 with CD177 (*i.e.*, the IgG is a *blocker*), then no further RU increase would be possible, but rather a decrease in RUs during flow of the complex, since bound CD177 will be lost from the immobilized IgG, and there is insufficient free CD177 in the complex mixture to take its place. To minimize the concentration of free CD177 in the complex sample, the concentration used was 10-fold higher than the PR3-binding affinity, as measured also by SPR ((7); Fig. 1*B*). Using this assay, we unambiguously identified three PR3 blockers among the tested IgGs (Fig. 1*C*, clones 7, 40, and 72), whereas the remainder were nonblockers (Table 1).

**Table 1:** Summary of the binding affinities and complex blocking abilities of the anti-CD177 monoclonal antibodies

Antibody (clone)	Affinity (×10^−9^ M)	ELISA (blocking capacity)	SPR (blocking capacity)
7-3-1	8.0	++	Yes
39-5-2	0.14	−	No
40-6-4	3.1	++	Yes
49-3-1	11.9	(+)	No
72-3-1	9.0	++	Yes
73-8-1	0.92	(+)	No
80-11-2	2.3	(+)	No
82-2-4	0.76	(+)	No
90-12-1	1.3	(+)	No
92-3-4	11.8	(+)	No

The ++ symbol indicates a clear blocking effect in ELISA experiments; − indicates no blocking effect; (+) indicates ambiguity as to a possible blocking effect.

### PR3 blocker and nonblocker IgGs and their corresponding Fab fragments bind to CD177^pos^ neutrophils, but only IgGs are activating

We next verified that our anti-CD177 monoclonal antibodies could bind to intact human CD177^pos^ neutrophils. Total neutrophil pools isolated from freshly donated blood samples were incubated with purified anti-CD177 IgG and subjected to fluorescence-activated cell sorting (FACS) analysis. Binding was confirmed in all cases (data not shown).

**Figure 2:**
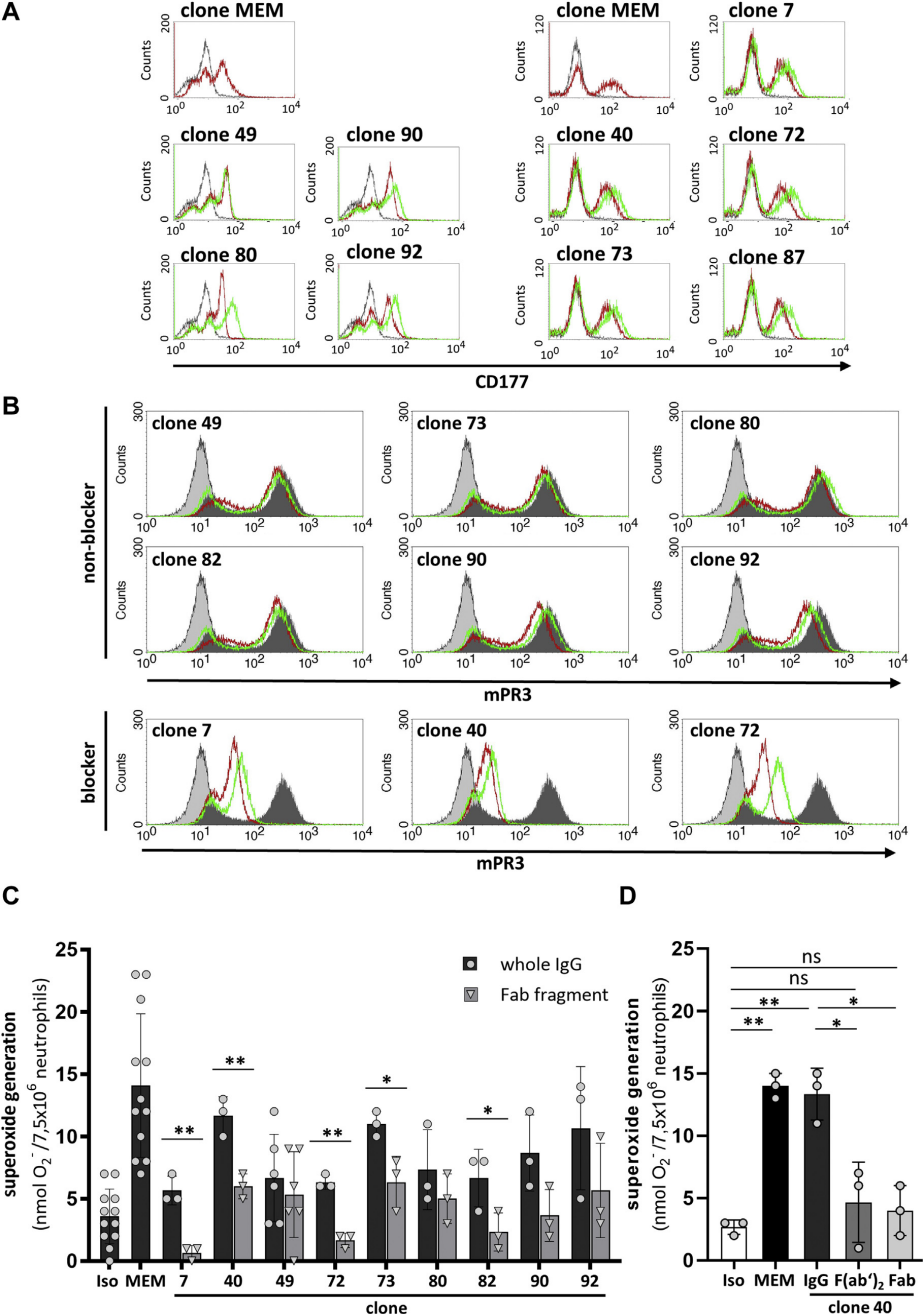
**Anti-CD177 Fab fragments bind to, but do not activate, CD177**^**pos**^**neutrophils.***A*, freshly isolated neutrophils were incubated with 5 μg/ml anti-CD177 IgG (*red line*) and anti-CD177 Fab (*green line*) followed by incubation with a FITC-conjugated goat antimouse IgG (Fab-specific (catalog no.: F5262; Sigma) secondary antibody. Isotype staining is shown in *gray*. Shown experiments were performed in two sets with two different human blood donors. *B*, neutrophils were primed with TNFα (2 ng/ml) and incubated with nonblocker or blocker anti-CD177 IgG or corresponding Fab (20 μg/ml), following incubation with anti-PR3-Alexa488 IgG (2.5 μg/ml). Histograms show isotype staining (*light gray*), mPR3 staining (*dark gray*), and the effect of anti-CD177 IgG (*red line*) and anti-CD177 Fab (*green line*) on the mPR3 staining accordingly. *C*, superoxide generation in neutrophils stimulated with anti-CD177 whole IgG (5 μg/ml) (*dark gray bars*) or corresponding Fab (5 μg/ml) (*light gray bars*). A non-CD177-binding isotype was used as negative control (Iso), a commercially available activating monoclonal IgG against CD177 (clone MEM) was used as positive control (MEM). *D*, superoxide generation in neutrophils stimulated with equimolar concentrations of anti-CD177 whole IgG (5 μg/ml) (*dark gray bars*), corresponding F(ab’)_2_ (3.67 μg/ml) (*middle gray bars*), and corresponding Fab fragments (1.66 μg/ml) (*light gray bars*), respectively. A non-CD177-binding isotype was used as negative control (*open bars*), a commercially available activating monoclonal IgG against CD177 (clone MEM) was used as positive control (MEM, *black bars*). Single experiment data and means ± SD are depicted (n = 3–12). Differences between groups were determined by multiple *t* test. ∗ indicates *p* < 0.05, ∗∗ indicates *p* < 0.01. IgG, immunoglobulin G; mPR3, membrane-bound proteinase 3; TNFα, tumor necrosis factor alpha.

We also tested purified Fab fragments derived from each IgG by papain digestion and, again, confirmed binding of each to CD177^pos^ neutrophils by FACS staining (Fig. 2*A*).

Next, neutrophils were preincubated with our anti-CD177 IgGs and/or Fab fragments to test if PR3 blockers (clones 7, 40, and 72) interfered with subsequent anti-PR3 IgG binding (Fig. 2*B*). In contrast to PR3 nonblockers, the PR3 blockers reduced the anti-PR3 staining signal in the CD177^pos^ neutrophil subset.

PR3 IgG-like PR3–ANCAs strongly activate neutrophils for respiratory burst, but their corresponding Fab fragments do not. We therefore tested whether our CD177 binders also produced this response. We incubated isolated primed neutrophils with either anti-CD177 IgG or their corresponding Fabs and then determined to what extent they initiated oxidative burst by measuring the production of superoxide in the resulting aliquots. Though to differing extents, in all cases, the multivalent anti-CD177 IgG provoked respiratory burst in mixed neutrophil pools (Fig. 2*C*). Their corresponding monovalent Fabs, however, showed no significant stimulatory effect, with most of the resulting superoxide concentrations comparable to the negative control value. These effects also showed no obvious correlation with either the blocking or nonblocking properties of the individual binders or their relative affinities to CD177. For all further experiments with neutrophils, we chose to proceed with the IgG blocker clone 40 (*K*_*d*_ = 3.1 × 10^−9^ M) and the nonblocker clone 80 (*K*_*d*_ = 2.3 × 10^−9^ M) since both binders have a CD177 affinity similar to that of PR3 (*K*_*d*_ = 4.1 × 10^−9^ M).

Previous work indicated that multivalent (Fab)_2_ fragments prepared from patient-derived PR3–ANCAs also activated neutrophils, whereas (Fab)_2_ fragments derived from murine anti-PR3 monoclonal IgG could not (16). We accordingly prepared (Fab)_2_ fragments from our murine blocker clone 40 by pepsin digestion and assessed their effects on mixed neutrophil pools. Although intact IgG did elicit superoxide release, the corresponding (Fab)_2_ did not show this stimulatory effect (Fig. 2*D*), consistent with the earlier study. These results potentially indicate a role for the Fc in this activation process.

**Figure 3:**
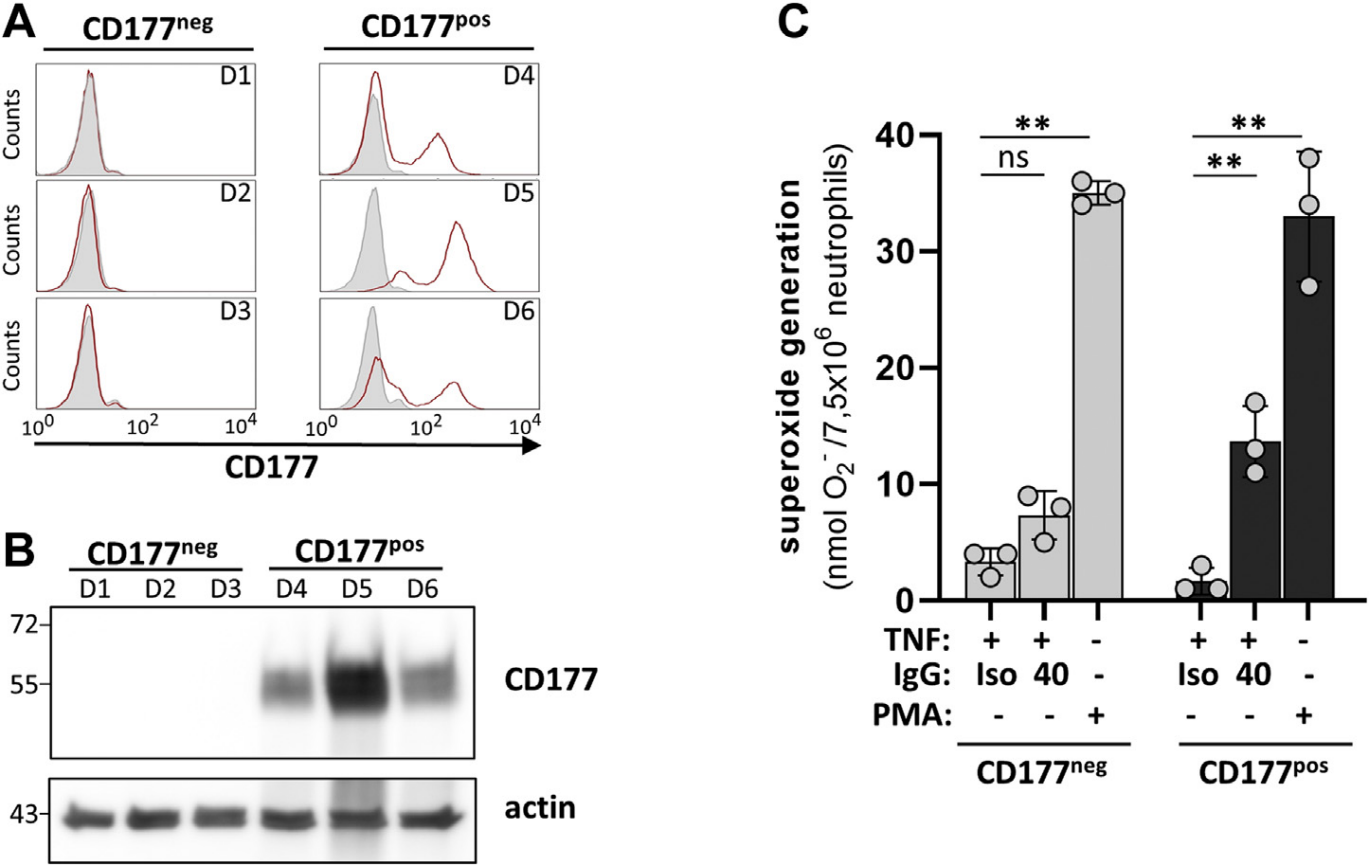
**Neutrophil activation by anti-CD177 IgGs requires CD177 binding.***A*, isolated neutrophils from CD177-deficient donors (D1–3) and CD177 bimodal individual donors (D4–6) were stained with isotype control (*gray*) or an anti-CD177 monoclonal antibody (mab) (*red*) and assessed by flow cytometry. *B*, neutrophil lysates from each donor were immunoblotted using an anti-CD177 antibody. Actin served as loading control. *C*, superoxide production of TNFα-primed neutrophils isolated from the CD177-deficient donors D1–D3 (*gray columns*) and the CD177 bimodal donors D4–D6 (*black columns*) after incubation with 5 μg/ml isotype control (iso), intact anti-CD177 mab clone 40, respectively. PMA stimulation served as positive control. Single data and mean ± SD are depicted. Comparison between treatment was done using one-way ANOVA and Tukey’s multiple comparison test, ∗∗ indicates *p* < 0.01. PMA, phorbol 12-myristate 13-acetate; TNFα, tumor necrosis factor alpha.

Using neutrophils from CD177-deficient donors, we confirmed that the observed activation effect required CD177 binding. We isolated neutrophils from three donors having no CD177^pos^ neutrophils by FACS staining, in parallel with three donors positive for CD177 showing the typical bimodal neutrophil distribution in FACS (Fig. 3*A*). We verified the FACS data by anti-CD177 immunoblotting of neutrophil lysates from each donor (Fig. 3*B*). We then repeated the activation assay with all neutrophil pools using intact blocker clone 40 IgG. As before, all three neutrophil pools from bimodal donors showed strong oxidative burst, whereas no substantial activation effect was seen in any pool from CD177-deficient donors (Fig. 3*C*).

### A PR3 blocking Fab reduces anti-PR3 IgG-stimulated oxidative burst in mixed (CD177^pos^/mPR3^high^/CD177^neg^/mPR3^low^) neutrophil populations

**Figure 4:**
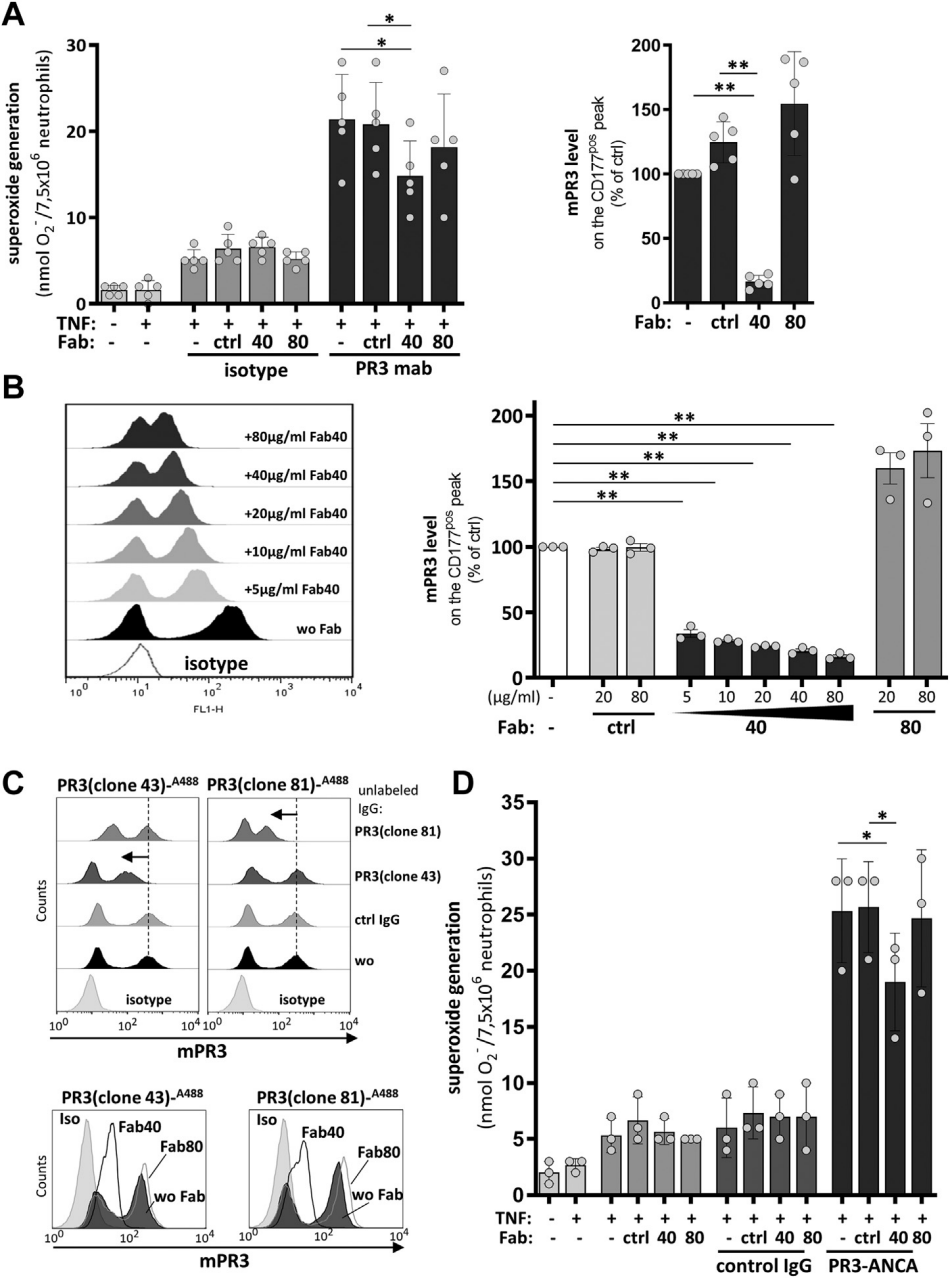
**Incubation with blocking anti-CD177 Fab reduces superoxide production in unsorted neutrophils.***A*, reduced superoxide production in unsorted neutrophils after incubation with blocking anti-CD177 Fab. Primed neutrophils were untreated or preincubated with 20 μg/ml control Fab (ctrl), blocker Fab (clone 40), or nonblocker Fab (clone 80), followed by 5 μg/ml stimulating monoclonal anti-PR3 IgG (*dark gray bars*), isotype control (*mid gray bars*), or without any further stimulation (*light gray bars*) (*left panel*, n = 5). In parallel, mPR3 staining of the CD177^pos^ neutrophil subset was assayed after incubation with Fabs as described (*right panel*, n = 5). *B*, dose-dependent reduction in mPR3 staining on neutrophils after incubation with blocking anti-CD177 Fab (clone 40). A representative set of histograms (*left panel*) and the corresponding geometric mean of mPR3 staining of the CD177^pos^ peak (*right panel*) from all experiments are shown. Untreated neutrophils were used as control. Non-CD177-binding control Fab (*light gray bars*), Fab clone 40 (*dark gray bars*), and nonblocking Fab clone 80 (*mid gray bars*) (n = 3). *C*, blocker Fab clone 40 negatively influences superoxide generation in neutrophils after PR3–ANCA stimulation (75 μg/ml;. (n = 3). Legend as in (*A*). Single experiment data and means ± SD are depicted. Comparison between multiple groups was done using one-way ANOVA and Tukey's multiple comparison test, ∗ indicates *p* < 0.05, ∗∗ indicates *p* < 0.01. ANCA, antineutrophil cytoplasmic autoantibody; IgG, immunoglobulin G; mPR3, membrane-bound PR3; PR3, proteinase 3.

We next tested whether preincubation of mixed population neutrophils with blocker Fab clone 40 had any effect on the stimulation of superoxide production by anti-PR3 IgG. We first incubated unsorted neutrophils with a saturating amount (20 μg/ml) of either blocker clone 40 or nonblocker clone 80 and, after washing, activated the neutrophils by addition of a monoclonal anti-PR3 antibody. The subsequent superoxide measurements showed that the anti-PR3 monoclonal IgG strongly stimulated the neutrophil pool in all cases, as opposed to treatment with an isotype IgG that provoked only a background response (Fig. 4*A*, *left panel*). While the degree of superoxide generation in the presence of nonblocker clone 80 was not significantly different than that measured in the absence of Fab or the presence of a non-CD177 binding control Fab, the pool containing blocker clone 40 showed a clearly weaker stimulation and correspondingly less superoxide production, implying that the PR3 blocking effect of clone 40 protects CD177^pos^ neutrophils from activation by anti-PR3 IgG.

FACS staining performed in parallel showed that pretreatment with blocker clone 40 significantly reduced anti-PR3 antibody binding to the CD177^pos^ neutrophil subset, whereas nonblocker and control Fab showed no reduction in mPR3 level (Fig. 4*A*, *right panel*).

To verify that the blocker clone 40 Fab was displacing CD177-bound PR3 from the CD177^pos^ neutrophils, we incubated unsorted neutrophils with increasing concentrations of clone 40 Fab and monitored changes in detectable mPR3 by FACS. In the absence of Fab, the peaks corresponding to the CD177^neg^/mPR3^low^ and CD177^pos^/mPR3^high^ populations were widely separated; upon addition of the clone 40 Fab, the separation between the peaks (stained for PR3) decreased dose-dependently until they merged, with very little difference distinguishable between them at the highest Fab concentration tested (Fig. 4*B*). We used two different anti-PR3 monoclonal IgGs recognizing two nonoverlapping PR3 epitopes (Fig. 4*C*, *left panel*) for detecting remaining PR3. Preincubation with blocker clone 40 Fab reduced the level of detectable PR3 as detected by both anti-PR3 IgGs, whereas nonblocker clone 80 did not. These observations confirmed that the blocker Fab 40 not only prevented PR3 binding to CD177 in SPR but also on the surfaces of living neutrophils. We then repeated our activation experiment with PR3–ANCAs obtained from AAV patient serum. As in the previous experiment with an anti-PR3 monoclonal IgG, only preincubation with blocker Fab 40 had a negative influence on the stimulation of superoxide production by PR3–ANCAs (Fig. 4*D*).

### The effects on neutrophil activation demonstrated by Fab blocker clone 40 are restricted to the CD177^pos^/PR3^high^ neutrophil population

**Figure 5:**
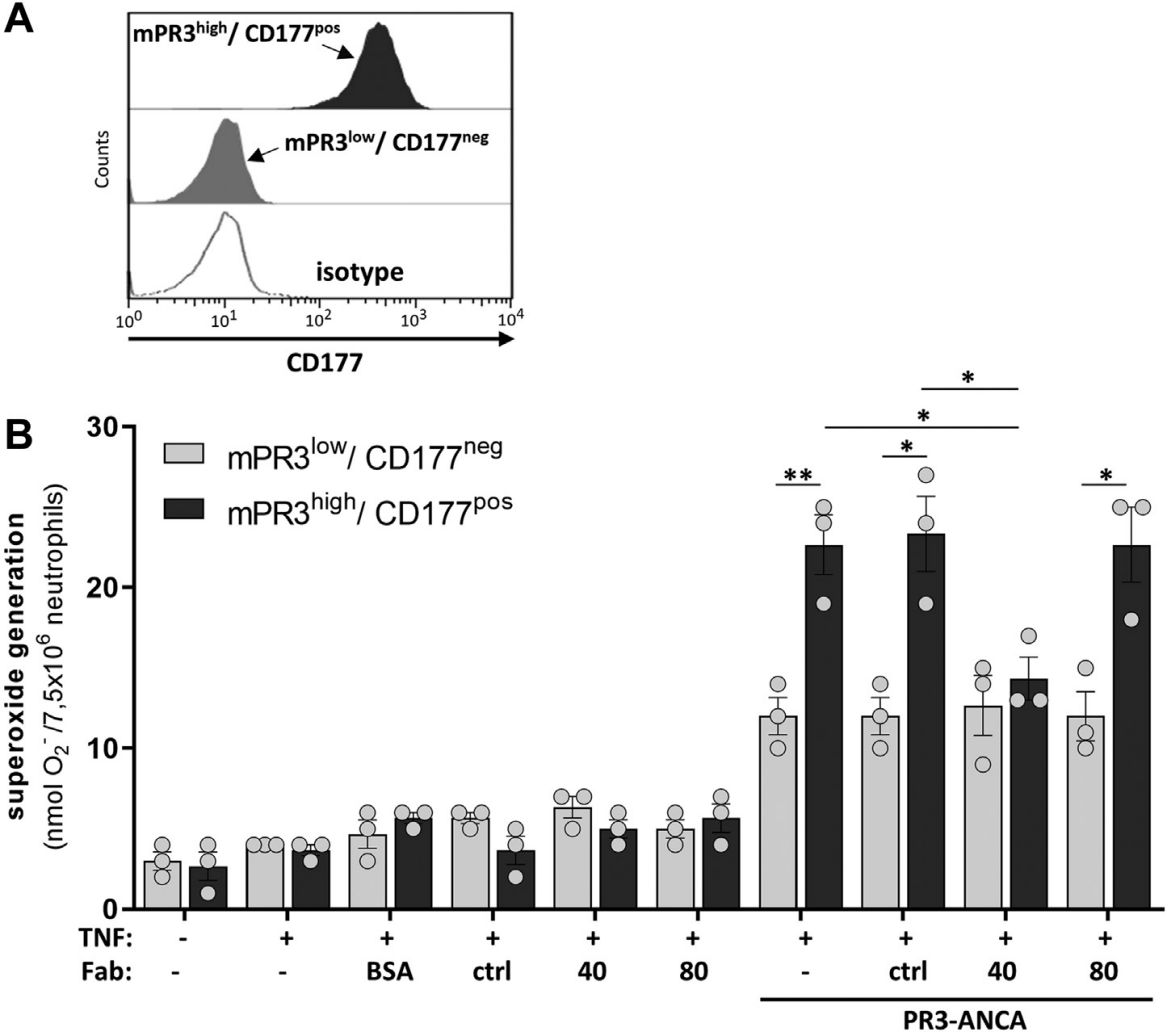
**Blocking anti-CD177 Fab only affects the activation of CD177**^**pos**^**/PR3**^**high**^**neutrophils.***A*, neutrophils from CD177/mPR3 bimodal donors were separated by magnetic cell sorting. Panel shows a representative separation after sorting into CD177^pos^/mPR3^high^ and CD177^neg^/mPR3^low^ subsets. *B*, blocking of neutrophil activation by anti-CD177 Fab clone 40 is restricted to the CD177^pos^/mPR3^high^ neutrophil subset. As described for Figure 3*A*, sorted neutrophils (CD177^pos^/mPR3^high^, *dark gray bars* and CD177^neg^/mPR3^low^, *light gray bars*) were assayed for superoxide generation. Because a PR3–ANCA epitope could be blocked by the monoclonal PR3 antibody used for the sorting procedure, polyclonal human PR3–ANCAs (75 μg/ml) were used for stimulation (n = 3). Single experiment data and means ± SD are depicted. Differences between groups were determined by multiple *t* test. Comparison between treatment was done using one-way ANOVA and Tukey’s multiple comparison test, ∗ indicates *p* < 0.05, ∗∗ indicates *p* < 0.01. ANCA, antineutrophil cytoplasmic autoantibody; mPR3, membrane-bound PR3; PR3, proteinase 3.

Preincubation of unsorted neutrophils with the PR3 blocking clone 40 Fab not only displaced PR3 from the CD177^pos^ population but also affected a clear reduction in the amount of superoxide produced as a result of stimulation with either monoclonal anti-PR3 IgG or PR3–ANCA IgG isolated from AAV patient serum. In order to more precisely define the effect of blocker clone 40, we repeated the PR3–ANCA stimulation experiments with sorted neutrophils. We used magnetic cell sorting with our isolated neutrophils to produce pure CD177^neg^/mPR3^low^ and CD177^pos^/mPR3^high^ preparations (Fig. 5*A*) and tested them separately by preincubation with 20 μg/ml blocker clone 40 Fab before washing and addition of the stimulatory PR3–ANCAs. As previously shown (15), both pure populations were activated by the addition of PR3–ANCAs, with the CD177^pos^/mPR3^high^ population producing nearly twice as much superoxide as the CD177^neg^/mPR3^low^ population (Fig. 5*B*). Preincubation with nonblocker clone 80 showed no effect on superoxide production; the values for both populations in the presence of this Fab were identical to those either without added Fab or preincubated with a control Fab. Preincubation with blocker clone 40 showed no measurable effect on superoxide production by stimulated CD177^neg^/mPR3^low^ neutrophils but a substantial effect with CD177^pos^/mPR3^high^ neutrophils. The presence of clone 40 completely eliminated the difference in superoxide production between the two pure neutrophil populations.

### Preincubation with Fabs does not affect cell viability

**Figure 6:**
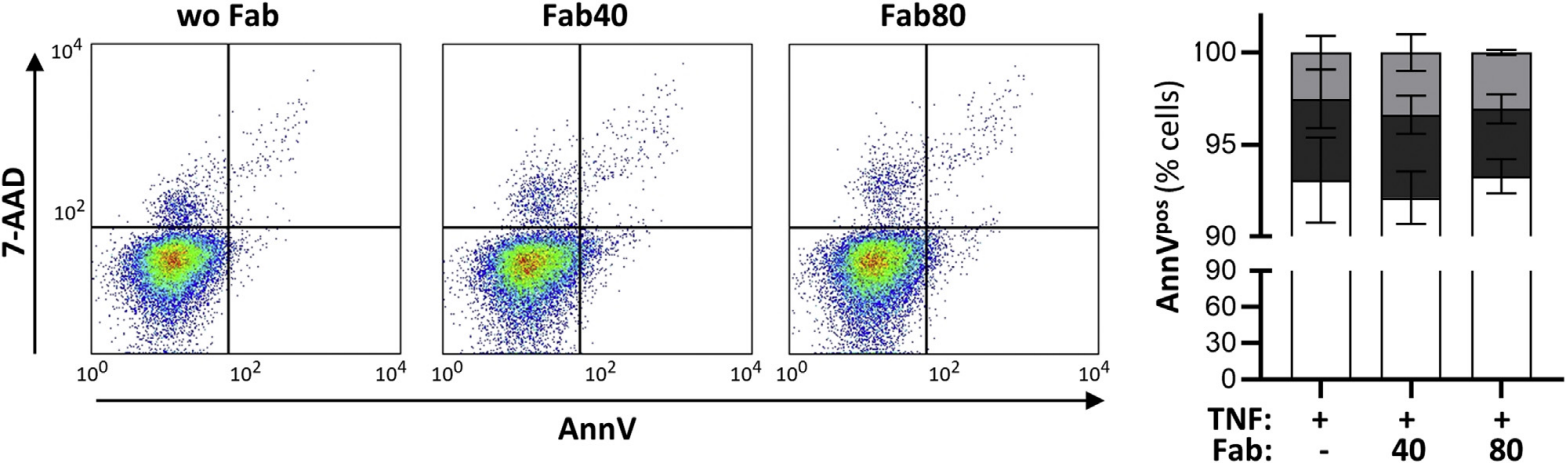
**Anti-CD177 Fabs do not influence neutrophil viability during incubation.** Isolated neutrophils were incubated with buffer control, clone 40, or clone 80 Fabs (each 20 μg/ml), respectively. After 2 h, cells were washed and double stained with annexinV/7-AAD. Typical histograms and the corresponding statistical analysis of the percentage of viable (annexinV^neg^/7-AAD^neg^, *open bar*), primary necrotic (annexinV^neg^/7-AAD^pos^, *dark gray bar*), and apoptotic (annexinV^pos^, *light gray bar*) cells is depicted (n = 3). 7-AAD, 7-actinomycin D.

To assess neutrophil viability during our experiments, we stained isolated neutrophils with necrosis and apoptosis markers with and without Fab incubation. Neutrophils were incubated alone, with blocker clone 40 Fab and with nonblocker clone 80 Fab for 2 h on ice. After incubation, washed cells were incubated with annexin V (Ann V; for detection of externalized phosphatidylserine, an indicator of apoptosis) and 7-aminoactinomycin D (7-AAD; a DNA intercalator, to determine membrane integrity that is lost during necrosis) and assessed by FACS. The staining results showed that >90% of the neutrophils remained viable for the duration of the incubations. No significant differences in either the number of apoptotic or necrotic cells was seen after incubation with either Fab, demonstrating that no blocker-induced decrease in superoxide generation was due to cell death (Fig. 6).

## Discussion

PR3 is a member of a family of neutrophil serine proteases important in inflammation and is known to process extracellular matrix proteins (3), cell surface receptors (16, 17), cytokines (18, 19), and intracellular effectors, including kinase inhibitors (20), cytoskeletal proteins (21), and Ann V (22). As a major component of azurophilic granules, it is also released in abundance from neutrophils during degranulation. Although it is a soluble protein, it is detectable on the extracellular surface of all neutrophils and readily adheres to the membranes of nonmyeloid cells as well (7). PR3 possesses a patch of hydrophobic residues on its surface that have been suggested to be responsible for its interaction with membranes (23), though definitive proof for this supposition has yet to be reported. To date, the only confirmed (nonsubstrate) interaction partner for PR3 is CD177, a glycosylphosphatidylinositol-anchored protein with no clearly defined function. Because of its high-affinity complex with PR3, CD177 is responsible for the abundant mPR3 occurring in neutrophils expressing it. As the target of autoantibodies, mPR3 is a central player in ANCA vasculitis that, in an as yet unknown manner, facilitates autoantibody-induced respiratory burst and degranulation, a process normally requiring multiple receptor-initiated signaling cascades (2). An outstanding question here is exactly how molecules that do not obviously function as receptor ligands and do not themselves cross the plasma membrane can nevertheless activate the intracellular signaling pathways necessary for the degranulation process. The fact that CD177^pos^ neutrophils react more strongly in this regard than CD177^neg^ neutrophils could be an indication that CD177—though not recognized by PR3–ANCAs—has, like PR3, a role in facilitating ANCA-initiated neutrophil activation, beyond serving as a platform for additional ANCA epitopes because of complexation with PR3. The experiments presented here address this possibility directly by blocking CD177:PR3 complex formation *in situ* and probing the effect of this epitope removal on the ANCA-induced activation response. The clear result was that once the bulk of the CD177-bound PR3 was titrated away from the neutrophil surface, the ANCA sensitivity of the CD177^pos^ population was fully reduced to that of the CD177^neg^ population. ANCA addition still elicited a respiratory burst from these neutrophils but this *via* the remaining directly membrane-bound mPR3 that exists in both populations. These results—along with the fact that CD177^neg^ neutrophils are also sensitive to ANCAs—make clear that CD177 is not required for the ANCA-induced activation effect. They also support the simplest explanation for the enhanced ANCA sensitivity of CD177^pos^ neutrophils, namely that the presence of CD177—likely all of which is bound to PR3—makes a larger abundance of ANCA epitopes available than is found on CD177^neg^ neutrophils. CD177 likely “presents” PR3 to the extracellular environment in a uniform orientation that maximizes accessibility to the most common PR3–ANCA epitopes (7). Our results imply that this increase in binding sites is sufficient to account for the increased ANCA sensitivity of these neutrophils, since physically removing them reduces ANCA sensitivity to that of CD177^neg^ neutrophils.

The results do not answer the question of what function(s) CD177 actually has in neutrophils and why it is found in complex with PR3. They also do not rule out entirely that CD177 can participate in the ANCA-induced activation process; the fact that anti-CD177 IgGs provoke a response similar to that of PR3–ANCAs provides clues for further studies into the role of CD177 in neutrophil biology. The newly developed antibodies described here will be of great value in analyzing the function of CD177 in such experiments and may also be of value in the treatment of AAV. Since the removal of CD177-bound PR3 results in a substantial reduction in ANCA-induced neutrophil activation, blocking this interaction *in vivo* could prove beneficial for PR3–AAV patients, particularly those with large CD177^pos^ neutrophil populations.

## Experimental procedures

### Hybridoma generation

Recombinant CD177 was prepared as described previously (7) and provided to Biogenes GmbH for inoculation of mice. Hybridomas delivered by Biogenes were cocultured on feeder cells obtained from peritoneal lavage of Black6 mice in hybridoma medium: Dulbecco's minimal essential medium (catalog no.: D5871; Sigma) supplemented with 20% fetal calf serum (Merck), 2 mM glutamine (Gibco), 1 mM sodium pyruvate (catalog no.: S8636; Sigma;), and antibiotics (penicillin/streptomycin; Gibco). All the IgGs used in this study were isotype IgG_1_.

### IgG isolation and Fab preparation

Stable hybridoma cultures were grown in 300 cm^2^ culture flasks (TPP) in 50 ml hybridoma medium. Cells were split 1:10 every 48 h (at 80–90% confluence) and with complete exchange into fresh medium. Collected medium aliquots containing IgG were pooled and passed over a 5 ml Protein G Agarose column (GE Healthcare) washed with PBS (Merck) and eluted with 50 mM glycine (Sigma), 150 mM NaCl (Sigma), pH 3.5 directly into 1 M Tris, pH 8.0 for neutralization. Elution fractions were pooled and concentrated in 100 kD molecular weight cutoff Amicon spin concentrators (Millipore). Concentrated IgGs were further purified over a Superdex 200 size-exclusion column (GE Healthcare) in 20 mM Hepes (Sigma), 150 mM NaCl, pH 7.4. IgG-containing fractions were verified by SDS-PAGE, pooled, concentrated in 30 kD molecular weight cutoff (MWCO) Amicons, and stored at 4 °C until use.

Fabs were prepared from purified IgG by incubation with Papain-Agarose (Thermo Fisher Scientific) according to the manufacturer’s instructions. Digested IgG was passed over Protein G agarose to remove Fc, and undigested IgG- and Fab-containing fractions were pooled, concentrated in 10 kD MWCO Amicons, and subjected to size-exclusion chromatography as for the IgG.

### (Fab)_2_ preparation

(Fab)_2_ fragments were prepared from purified IgG by incubation with immobilized pepsin (Pepsin–Agarose; Sigma) according to the manufacturer's instructions. After digestion, the reaction mixture was centrifuged at 300*g* to pellet the pepsin–agarose. The solution was removed, neutralized by addition of 1 M Hepes, pH 7.4, concentrated in 30 kD MWCO Amicons, and subjected to size-exclusion chromatography as for the IgG.

### SPR experiments

Experiments were performed on a ProteOn XPR36 instrument (Bio-Rad) using standard amine chemistry for coupling IgG to the sensor chip (GLH sensor chips; Bio-Rad). Ligand dilution series were prepared in ProteOn running buffer (PBS supplemented with 0.005% Tween-20 [Sigma]).

### Neutrophil preparation

Blood neutrophils from healthy and AAV patient donors were obtained (Ethic Votum EA1/277/11, approved by the Ethics Commission of the Charité, Berlin, in accordance with the principles of the Declaration of Helsinki) and purified as described previously (19). Briefly, neutrophils from healthy volunteers were isolated from heparinized whole blood by red blood cell sedimentation with 1% dextran, followed by Histopaque 1.083 (Sigma) density gradient centrifugation, and hypotonic erythrocyte lysis. Neutrophils were centrifuged and resuspended in Hank's balanced salt solution with calcium and magnesium (Hank's balanced salt solution++; Merck). Cell viability was determined by Trypan blue exclusion and exceeded 99%. PR3–ANCA IgG was prepared from the plasmapheresis fluid of a single AAV patient, a 29-year-old male with active PR3–ANCA granulomatosis with polyangiitis, with ear, nose, and throat, eye, lung, and crescentic glomerulonephritis disease manifestations. The use of residual material, such as plasmapheresis fluid, was covered by hospital regulations at admission with no requirement of informed consent.

### Separation of CD177^pos^/mPR3^high^ and CD177^neg^/mPR3^low^ neutrophil subsets by magnetic beads

Neutrophil subsets were separated with magnetic-activated cell sorting (MACS) separation columns (Miltenyi Biotec). Isolated neutrophils were stained with monoclonal anti-PR3 (clone 43-8-3-1). MACS rat antimouse IgG1 beads were added, and cells were pipetted onto a MACS LD column, and the flowthrough containing the nonlabeled CD177^neg^/mPR3^low^ neutrophils was collected. Columns were removed from the magnet to allow collection of the labeled CD177^pos^/mPR3^high^ cells. The purity of the two separated subsets was assessed by flow cytometry using a FITC-labeled anti-CD177 IgG.

### Membrane PR3 expression on neutrophils

Neutrophils were stimulated with 2 ng/ml tumor necrosis factor alpha (TNFα) (30 min, 37 °C; R&D Systems) to increase the amount of membrane PR3. Cells were washed and stained with monoclonal anti-PR3 (clone 81-3-3)-Alexa488-conjugated IgG (2.5 μg/ml; 20 min on ice). mPR3 expression was assessed by flow cytometry using a FACSCalibur instrument. Ten thousand events per sample were assayed.

For blocking experiments, TNFα-primed neutrophils were incubated with 20 μg/ml anti-CD177 IgG or Fab (60 min, on ice). The capacity to block anti-PR3 IgG binding was tested by subsequent incubation with the Alexa488-conjugated anti-PR3 IgG.

### Measurement of respiratory burst

Superoxide was measured using the assay of superoxide dismutase–inhibitable reduction of ferricytochrome c. Neutrophils were pretreated with 5 μg/ml cytochalasin B for 15 min on ice. Cells (0.75 × 10^6^/ml) were primed with 2 ng/ml TNFα for 15 min before stimulating antibodies (or Fabs) were added. The final concentration was 5 μg/ml for monoclonal antibodies or Fabs.

For superoxide blocking experiments, cells were incubated with 20 μg/ml or with indicated amounts of anti-CD177 Fab during the TNFα priming, before stimulating monoclonal anti-PR3 (clone 43-8-3-1) or 75 μg/ml purified PR3–ANCA preparations were added.

Experiments were performed in 96-well plates at 37 °C for up to 45 min, and the absorption of samples with and without 300 U/ml superoxide dismutase was measured at 550 nm using a microplate reader (Molecular Devices).

### Flow cytometry Ann V/7–AAD staining

To monitor phosphatidylserine externalization and cell death, 10^6^ neutrophils were washed in ice-cold PBS and resuspended in 200 μl ice-cold Ann V binding buffer (10 mM Hepes, pH 7.4, 140 mM NaCl, 2.5 mM CaCl_2_) and incubated with FITC-Ann V (BD Biosciences; 1:100 dilution) for 15 min at room temperature protected from light. 7-AAD (Thermo Fisher Scientific; final 1 μg/ml) was then added. Analysis was performed within 1 h.

### Statistical analyses

We used Prism 8.4.3 (GraphPad Software, Inc) and performed multiple *t* tests and one-way ANOVA with Tukey's post hoc test. ∗ indicates *p* < 0.05 and ∗∗*p* < 0.01.

## Data availability

All data generated or analyzed during this study are included in this published article.

## Conflict of interest

The authors declare that they have no conflicts of interest with the contents of this article.
